# Partial response of liver metastases treated with abiraterone in castration-resistant prostate cancer: A case report

**DOI:** 10.3892/ol.2013.1275

**Published:** 2013-03-29

**Authors:** ILARIA MARECH, ANGELO VACCA, NICOLA SIVESTRIS, ANTONIO GNONI, VITO LORUSSO

**Affiliations:** 1 Department of Biomedical Sciences and Human Oncology, University of Bari Medical School, Bari;; 2 Medical Oncology Unit, National Cancer Research Centre, Giovanni Paolo II Tumor Institute, Italy; 3 Medical Oncology Unit, Vito Fazzi Hospital, Lecce, Italy

**Keywords:** abiraterone, castration-resistant prostate cancer, visceral metastases, hormonal treatment

## Abstract

Docetaxel is the current first-line treatment for castration-resistant prostate cancer (CRPC), following failure to respond to maximal androgen blockade (MAB). Patients who fail to respond to docetaxel may receive cabazitaxel or abiraterone; however, there are no recommendations on which of these two agents should be used first. Here, we present a case of a male patient suffering from CRPC with liver and lymph node metastases, in which abiraterone achieved a partial response, according to RECIST criteria. In the literature, visceral involvement in patients with advanced prostate cancer is an infrequent occurrence; it affects 18–22% of patients. In the pivotal study concerning docetaxel-resistant patients, abiraterone was compared with a placebo and the forest plot for survival demonstrated that patients with visceral involvement have significantly benefited from abiraterone. In the TROPIC trial comparing cabazitaxel with mitoxantrone, the proportion of patients with visceral disease was ∼25% in both arms and there was no difference in overall survival in this subgroup of patients. In our case, we observed a significant activity of abiraterone in lymph node and liver metastases. If confirmed in large studies, this observation may raise concerns over whether to treat patients suffering from CRPC and visceral metasis with chemotherapy or hormone therapy.

## Introduction

Prostate cancer is the most common type of cancer in males in Western countries and the USA (1,2). The key first-line treatment of advanced prostate cancer is maximal androgen blockade (MAB). MAB is obtained with a combination of LHRH analogs and an antiandrogen active against androgen receptors (AR), and is known as bicalutamide (androgen deprivation therapy; ADT) (3). However, despite ADT, disease progression is a frequent and predictable event in the natural history of prostate cancer (3–5).

In castration-resistant prostate cancer (CRPC) patients, docetaxel became the standard first-line treatment. Docetaxel was approved by the US Food and Drug Administration (FDA) in 2004, as it demonstrated a significant improvement in overall survival, median time to progression and prostate-specific antigen (PSA) reduction (4,5) compared with mitoxantrone.

Currently, patients who fail to respond to docetaxel treatment may receive either second-line chemotherapy with cabazitaxel (a recent semi-synthetic taxoid), or second-line hormonal treatment with abiraterone acetate (a new anti-androgen, which demonstrated high efficacy in docetaxel pre-treated patients) (6,7).

Abiraterone acetate is a selective, potent oral small-molecule inhibitor of cytochrome P17, which catalyses two key reactions (17-α-hydroxylase and 17,20-xylase), interfering with adrenal androgen synthesis (8,9) and thereby dramatically reducing adrenal, testicular and intra-tumoural androgen production. The pivotal phase III trial (NCT00638690) conducted on 1,195 patients (7) that had been previously treated with a docetaxel-based regimen, demonstrated an improvement in overall survival of 4 months in the abiraterone arm as compared with the placebo. Moreover, abiraterone also had an advantage in terms of PSA response rate (28%), time to PSA progression (3 months) and radiographic progression-free survival (R-PFS; 2 months).

Abiraterone has demonstrated a favourable safety profile with a low frequency of adverse events, which were predominantly of grade 1 or 2. The most common side effects were fluid retention (31%), hypokalemia (17%) and hypertension (10%). These were secondary to mineral corticoid excess, resulting from a blockade of CYP17, which was abrogated by low-dose corticosteroids. Other common adverse events, not correlated with mineral-corticoid excess, were fatigue (44%), back and bone pain (30–25%), nausea (30%), arthralgia (27%) and anemia (23%) (7).

Here we report the case of a male patient with metastatic (bone, liver and retroperitoneal lymph nodes) CRPC, who was evaluated as demonstrating a partial response according to Response Evaluation Criteria in Solid Tumors (RECIST) (10), following treatment with abiraterone. The study was approved by the ethics committee of the Ospedale Vito Fazzi ASL LE, Lecce, Italy, and written informed consent was obtained from the patient before study entry.

## Case report

The patient was a 65-year-old male who presented in May 2007 with thalassemia trait and idiopathic hypertension. He had been previously subjected to an appendicectomy and was complaining of back pain. Haematological examination revealed an increased PSA level of 509 ng/ml. The prostate appeared uniformly hypo-echoic with no evidence of any lesion in the transrectal ultrasound scan. However, a biopsy performed on May 27th 2007 revealed an adenocarcinoma of the prostate with a Gleason score of 9 (5+4). In addition, on August 8th 2007, a bone scan revealed metastatic bone lesions in the thoracic and lumbar vertebrae (D9, D12, L2 and L4). The patient underwent antalgic radiotherapy on the lumbar spine (30 Gy in 15 fractions), which slightly reduced the pain.

Systemic therapy was initiated with the LHRH inhibitor leuprorelin acetate (Enantone) at a dose of 3.75 mg subcutaneously every 28 days, as well as bicalutamide (Casodex) 50 mg daily and orally. During maximal androgen blockade (MAB), the PSA level fell to a nadir of 80 ng/ml. After eight months of treatment, the patient presented a recurrence of back pain and biochemical failure, revealed by an increase in the PSA value to 180 ng/ml. A bone scan performed in January 2008 revealed an increased uptake of technetium-99m, as compared with the previous bone scan. Additionally, new lesions (lumbar vertebrae L1 and L3) were observed. The serum value of testosterone was at castration level; 20 ng/ml. The patient was crossed over to antiandrogen withdrawal, which produced no effect on either the PSA level or on the patient’s pain. The patient continued on leuprolide and, in February 2008, chemotherapy with docetaxel at a dosage of 75 mg/mq every three weeks was initiated, in association with zoledronic acid every 28 days. The patient demonstrated a good tolerability to this regimen, exhibiting a clinical benefit (disappearance of pain), PSA response (10 ng/ml from the basal 200 ng/ml) and radiologically stable disease (no new bone metastases were evident on a CT scan performed after three months of therapy).

**Figure 1:**
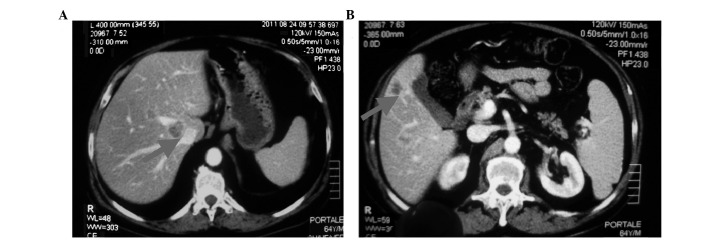
Computerised tomography (CT) scan performed on August 24th 2011, demonstrating liver metastases with maximum diameters of 3 cm (A) and 2 cm (B).

Therefore, docetaxel was continued until August 2011, when a whole-body CT scan revealed involvement of mediastinal and retroperitoneal nodes (hepatic hilum, para-caval, inter-aortocaval, para-aortic and iliac bilaterally) with a maximum diameter of ∼5 cm, and multiple liver lesions with a maximum diameter of ∼3 cm (Fig. 1). Another bone scan was performed, revealing an additional bone lesion on the 7th left rib. Also, biochemical progression was observed; the PSA level rapidly increased to 500 ng/ml. However, the serum level of testosterone remained at 10 ng/ml.

**Figure 2:**
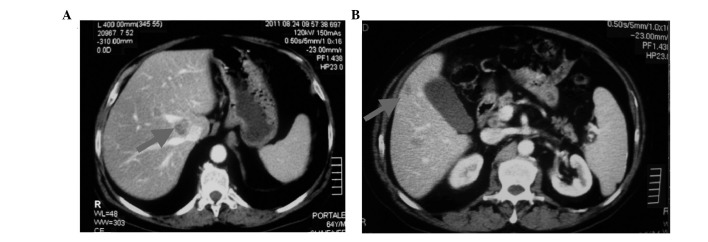
Computerised tomography (CT) scan performed on December 10th 2011, demonstrating a partial response of liver metastases with maximum diameters of 1.7 cm (A) and 1 cm (B).

Based on the evidence that the disease was progressive, chemotherapy was interrupted, and a second-line hormonal therapy was started in October 2011 with abiraterone acetate at a dosage of 1000 mg/day orally, along with 10 mg prednisone. Following treatment for 1 month, the PSA level was reduced to 350 ng/ml. A CT scan of the abdomen and pelvis (performed on December 15th 2011) revealed a significant reduction in the retroperitoneal nodes and liver lesions; the maximum diameter of the nodes was 2.5 cm, as compared with 5 cm in the basal examination, and that of the lesions was 1.8 cm, as compared with 3 cm in the basal examination (Fig. 2). However, bone metastasis and the mediastinal nodes were unchanged. Moreover, a further reduction in the PSA level to 190 ng/ml was observed. The treatment was well-tolerated and the serum biochemistry was normal, except for hyperglycemia (between 130 and 180 mg/dl). In addition, the patient’s pain was greatly reduced.

The patient continued to receive abiraterone acetate and zoledronic acid, and remained asymptomatic on this drug combination, with PSA levels of 140 ng/ml. Two consecutive CT scans performed on February 24th and May 31st 2012 confirmed the partial remission of liver lesions and retroperitoneal lymph nodes. However, a bone scan, performed in May 2012, revealed the appearance of two new lesions, which together with the increase of PSA (240 ng/ml) led us to consider the patient as being in progression. The patient is currently being treated with cabazitaxel.

## Discussion

Treatment of advanced CRPC has dramatically improved in recent years, due to the availability of new chemotherapeutic agents, including cabazitaxel, and of new antiandrogens, such as abiraterone. It has been demonstrated that prostate cancer maintains a certain degree of hormonal dependence even following failure to respond to treatment with MAB. In particular, new antiandrogens, including abiraterone, interfere with adrenal, testicular and intra-tumoural androgen synthesis, (in addition to blocking AR) (3,8,9).

With regard to the choice of therapy following docetaxel failure, there are no guidelines or recommendations concerning which of the two active agents currently available, cabazitaxel or abiraterone, should be used first. The presence of visceral metastases may be an important consideration when selecting second-line therapy following docetaxel application, given the higher activity of chemotherapy compared with hormonal treatment in other diseases, such as breast cancer.

It is noteworthy that in patients with advanced prostate cancer, visceral involvement is not frequent. In fact, in the SWOG 99-16 and TAX 327 studies, the majority of patients had only bone disease (80–90%), whereas visceral involvement was present in only 18–22% of patients (4,5).

The NCT00638690 phase III trial, comparing abiraterone with a placebo in docetaxel-resistant patients, enrolled 11% of patients with liver metastases in an abiraterone arm and 8% in a placebo arm. Although the authors did not reveal the response rate to abiraterone in this subgroup of patients, the forest plot for survival demonstrated that these patients significantly benefited from abiraterone rather than the placebo (7).

In the TROPIC trial, the number of patients with visceral disease was not higher than 25% in either the cabazitaxel or mitoxantrone arms. Additionally, the response rate to visceral metastases was not revealed. However, the authors demonstrated that there was no difference in the overall survival of patients with visceral metastases between cabazitaxel- or mitoxantrone-treated patients (6).

In the present case, we observed a significant reduction of liver and node metastasis in a patient who was resistant to docetaxel and was treated with abiraterone. Moreover, the clinical partial response, according to RECIST criteria (10), was achieved relatively quickly, as demonstrated by the first re-evaluation CT scan performed after treatment for two months. Notably, the patient demonstrated a reduction in the level of PSA after one month (from 500 to 350 ng/ml). In addition, the response obtained in the visceral disease was maintained for seven months and the disease was only considered to be in progression due to the rising PSA level and the appearance of two new lesions during the bone scan, while the liver and lymph node lesions consistently exhibited shrinkage, as compared with the basal measurement. This demonstrated a high activity of abiraterone also in visceral metastasis.

As a final consideration, we highlight that our knowledge of the correct therapy sequence in patients with metastatic prostate cancer is still not complete. It has been demonstrated previously that abiraterone may also be effective in patients who are chemo-naive (11). Conversely, Mezynski *et al* suggested that abiraterone may be more active in patients pre-treated with docetaxel chemotherapy. This assumes that there is a cross-resistance between abiraterone and docetaxel, based on the interference of high intra-tumoural androgen level and observed after abiraterone withdrawal, which may interfere with docetaxel activity. However, this was a retrospective analysis based on the PSA response of few patients (12).

In view of the conflicting results of these preliminary studies, it is mandatory to conduct further studies to establish the correct and effective order of abiraterone treatment; before or after docetaxel.
